# A Stained-Free Sperm Morphology Measurement Method Based on Multi-Target Instance Parsing and Measurement Accuracy Enhancement

**DOI:** 10.3390/s25030592

**Published:** 2025-01-21

**Authors:** Miao Hao, Rongan Zhai, Yong Wang, Changhai Ru, Bin Yang

**Affiliations:** 1 Research Center of Robotics and Micro Systems, School of Mechanical and Electrical Engineering, Soochow University, Suzhou 215021, China; 20194029006@stu.suda.edu.cn; 2 School of Mechatronic Engineering and Automation, Shanghai University, Shanghai 200444, China; zra123@shu.edu.cn; 3 School of Electronic and Information Engineering, Suzhou University of Science and Technology, Suzhou 215009, China; wangyong@mail.usts.edu.cn (Y.W.); rch@usts.edu.cn (C.R.); 4 The First Affiliated Hospital of Soochow University, Suzhou 215129, China

**Keywords:** stained-free sperm, non-invasive, morphological analysis

## Abstract

Sperm morphology assessment plays a vital role in semen analysis and the diagnosis of male infertility. By quantitatively analyzing the morphological characteristics of the sperm head, midpiece, and tail, it provides essential insights for assisted reproductive technologies (ARTs), such as in vitro fertilization (IVF). However, traditional manual evaluation methods not only rely on staining procedures that can damage the cells but also suffer from strong subjectivity and inconsistent results, underscoring the urgent need for an automated, accurate, and non-invasive method for multi-sperm morphology assessment. To address the limitations of existing techniques, this study proposes a novel method that combines a multi-scale part parsing network with a measurement accuracy enhancement strategy for non-stained sperm morphology analysis. First, a multi-scale part parsing network integrating semantic segmentation and instance segmentation is introduced to achieve instance-level parsing of sperm, enabling precise measurement of morphological parameters for each individual sperm instance. Second, to eliminate measurement errors caused by the reduced resolution of non-stained sperm images, a measurement accuracy enhancement method based on statistical analysis and signal processing is designed. This method employs an interquartile range (IQR) method to exclude outliers, Gaussian filtering to smooth data, and robust correction techniques to extract the maximum morphological features of sperm. Experimental results demonstrate that the proposed multi-scale part parsing network achieves 59.3% $AP_{\mathrm{vol}}^{p}$, surpassing the state-of-the-art AIParsing by 9.20%. Compared to evaluations based solely on segmentation results, the integration of the measurement accuracy enhancement strategy significantly reduces measurement errors, with the largest reduction in errors for head, midpiece, and tail measurements reaching up to 35.0%.

## 1. Introduction

**Figure 1:**
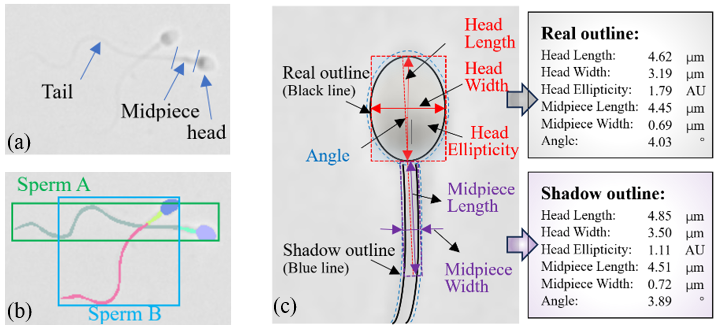
Sperm morphology segmentation and measurement. (**a**) Sperm structure components: head, midpiece, and tail. (**b**) Multi-sperm instance parsing distinguishes individual sperm and analyzes their structure. (**c**) Low-resolution sperm images cause measurement errors, as blurred boundaries (black vs. blue lines) lead to contour inaccuracies, significantly impacting morphology parameters.

Sperm morphology is closely associated with sperm function and is widely recognized as a critical indicator of sperm quality [1,2]. The measurement of sperm morphology [3] involves the quantitative analysis of size and shape parameters of the sperm head, midpiece, and tail (as shown in Figure 1a) using microscopy or other imaging techniques. This approach has extensive applications in both fundamental research and clinical settings. In assisted reproductive technologies (ARTs), such as in vitro fertilization (IVF), sperm morphology measurement plays a pivotal role by enabling the selection of morphologically superior sperm [4]. This, in turn, significantly enhances fertilization rates and improves embryo development quality. Despite its importance, current sperm selection methods primarily rely on qualitative assessments by embryologists, which are subjective and often inconsistent due to their dependence on individual expertise [5]. Additionally, traditional sperm morphology analysis often involves staining, which can damage the sperm and is unsuitable for use in settings such as IVF. To address these limitations, there is a pressing need for an automated, accurate, and non-invasive sperm morphology measurement method. Such a method would provide a more objective and quantitative tool, facilitating the reliable identification of optimal sperm for use in intracytoplasmic sperm injection (ICSI) and further advancing the precision of ART practices.

Automated and non-invasive sperm morphology assessment heavily relies on accurate part segmentation and morphological evaluation of each non-stained sperm (see Figure 1a). However, two major challenges need to be addressed to achieve this goal: (1) Instance parsing for multiple sperm targets. In ICSI procedures, selecting the sperm with optimal morphology and motility from a large pool of samples is essential. According to the WHO manual, sperm morphology assessment requires detailed observation and measurement of the head, midpiece, and tail. However, conventional instance or semantic segmentation methods are insufficient for multi-sperm morphology evaluation. Thus, instance parsing for multiple sperm targets (as shown in Figure 1b) is crucial, enabling not only precise distinction of individual sperm but also fine-grained segmentation of the head, midpiece, and tail to extract comprehensive morphological features. (2) Eliminating measurement errors caused by reduced resolution in non-stained sperm images. Traditional morphology assessment relies on fixation and staining techniques, which enhance recognition through color contrast but damage sperm and their DNA, making them unsuitable for clinical use. To prevent sperm from swimming out of the field of view under high-magnification objectives, sperm selection is typically performed under a 20× magnification. However, the reduced resolution of low-magnification images results in blurred boundaries and loss of details, which affect the accuracy of segmentation methods. As shown in Figure 1c, blurred boundaries lead to discrepancies between the true contour (black line) and the shadow contour (blue line), further impacting the accuracy of morphological parameters. For example, a normal head length (3.7–4.7μm) might be incorrectly calculated as 4.85μm due to shadow-induced errors, resulting in the misclassification of a normal head as abnormal.

In automated and non-invasive sperm morphology assessment, the primary challenge is accurately parsing multiple sperm targets. Advances in computer vision and deep learning [6,7] have significantly improved sperm segmentation [8,9], but previous methods focused mainly on semantic or basic instance segmentation [10,11,12]. These methods did not address the need for distinguishing and parsing multiple sperm instances for detailed morphology measurements. Large segmentation models [13,14,15], based on Transformer architectures and utilizing self-attention mechanisms, excel in capturing both global and local features and are widely applied in the segmentation of natural images. However, these models focus on global patterns and the handling of large objects, lacking the fine-grained detail capture required for accurate sperm instance parsing. Additionally, their high computational complexity limits their applicability in real-time tasks. Therefore, although these models perform exceptionally well in general object segmentation tasks, they are not well suited for sperm instance parsing. As a result, instance-level parsing of multiple sperm targets has become a critical step in morphological measurement [16]. This process requires not only accurately separating each sperm instance from the background but also extracting and analyzing detailed features for each instance to enable subsequent morphological analysis [17,18]. Currently, state-of-the-art methods for multi-target instance parsing are mostly applied to human parsing [19,20,21], and can be categorized into top-down and bottom-up strategies [22]. Top-down methods [23,24] detect target regions first and refine local features progressively. However, due to the focus on global information during feature extraction, large-scale features tend to overlook small-scale details [6]. This is especially true when using large convolution kernels or fewer sampling points, leading to the blurring or loss of details for small targets [25], such as sperm tails. In contrast, bottom-up methods [20,26] start by segmenting pixels and then aggregating them into object instances, capturing local details more precisely but lacking global structural constraints, which may lead to incomplete parsing of small targets like sperm parts.

After segmentation, addressing measurement errors caused by low-resolution non-stained sperm images is essential for accurate morphology evaluation. Key parameters of the sperm head, midpiece, and tail are highly sensitive to such errors (e.g., the head’s length, width, etc.) [27,28], with minor deviations causing significant inaccuracies (see Figure 1c). Currently, methods to improve measurement accuracy under low-resolution conditions can be broadly classified into three categories: super-resolution reconstruction, multi-frame image fusion, and image enhancement techniques. Super-resolution methods, such as SRCNN [29] and ESRGAN [30], improve image details but are computationally intensive and unsuitable for real-time analysis. Multi-frame fusion generates high-resolution images by aligning and merging multiple frames but requires precise registration and is time-consuming. Image enhancement techniques, like Gaussian filtering and histogram equalization, improve edge clarity and segmentation accuracy but risk losing details or amplifying noise, compromising measurement reliability.

To address the challenges, we propose a novel non-stained sperm morphology measurement method integrating a multi-scale part parsing network with measurement accuracy enhancement. The network combines instance and semantic segmentation to achieve instance-level parsing for sperm morphology. The instance segmentation branch creates masks for accurate sperm localization, while the semantic segmentation branch provides detailed segmentation of sperm parts. Outputs from both branches are fused for comprehensive parsing. To reduce measurement errors from low-resolution images, we propose a method based on statistical analysis and signal processing, including outlier filtering, Gaussian smoothing, and maximum value extraction. Experimental results show a 59.3% $AP_{\mathrm{vol}}^{p}$ on the dataset, outperforming AIParsing by 9.20%, and reducing measurement errors in sperm head, midpiece, and tail parameters by up to 35.0%.

## 2. Related Works

This section reviews related studies on multi-sperm target instance parsing, with a particular focus on multi-scale part parsing techniques based on the fusion of semantic and instance segmentation. Subsequently, existing methods for measurement accuracy enhancement are discussed.

### 2.1. Multi-Target Instance Parsing

Currently, the inspiration for multi-sperm target instance parsing often stems from instance parsing in human body analysis, which involves segmenting human parts (e.g., arms, legs, and hair) and associating each segmented part with its corresponding instance. State-of-the-art instance-level human parsing solutions can be categorized into two main approaches: top-down and bottom-up. Top-down approaches [22,23,24,31] treat instance-level human parsing as a global fine-grained semantic segmentation task. These methods start by detecting and segmenting human regions from the overall image and then progressively refine the segmentation to parse individual human parts. They rely heavily on global feature information to first identify the rough contours of human instances and subsequently extract detailed information for each part. For example, AIParsing [22] employs an anchor-free detector (FCOS) to achieve pixel-level instance localization. It uses an edge-guided parsing branch to refine the segmentation of each detected instance, effectively distinguishing different human body parts. Parsing R-CNN [31], built upon the classical instance segmentation network Mask R-CNN, first uses an instance segmentation module to extract region of interest (ROI) masks for targets and then performs part parsing within each ROI. This method further divides each instance into specific parts (e.g., head, torso), enabling multi-target parsing. Self-Correction for Human Parsing (CE2P) [23] integrates context embedding and edge-aware modules to enhance parsing precision by capturing global features and refining boundaries. High-Resolution Network (HRNet) [24] maintains high-resolution feature maps throughout the network to effectively preserve image details. Its core architecture consists of multiple branches processing features at different resolutions, which are fused at each stage with high-resolution branches. This design combines local and global information, allowing HRNet to retain fine details while accommodating multi-scale targets. However, top-down methods rely on the results of global detection to constrain their segmentation range and feature extraction region. If the global detection fails to fully encompass the target region (e.g., the detection box does not entirely cover the target), certain local regions may be omitted or incorrectly segmented, leading to reduced segmentation accuracy. Bottom-up approaches [20,26,32], on the other hand, focus on local regions first and progressively build the overall parsing results from local information (e.g., key points or small regions). These methods typically depend on local feature information and combine these features to generate complete instance parsing results. Part Grouping Network (PGN) [20] begins by segmenting individual parts of the human body using a grouping strategy, treating the parsing task as a node relationship modeling problem in a graph structure. Information propagation mechanisms are then employed to integrate global and local information before merging to achieve instance-level parsing. Graphonomy [32] uses graph neural networks to model different human parts as nodes in a graph structure. Through information propagation between nodes, it achieves detailed instance parsing and global optimization. This method emphasizes the integration of local features and global information, enhancing the parsing of different parts and improving the model’s generalization performance. DeepLab [26] introduces conditional random fields (CRFs) to refine the network’s output. By modeling relationships between pixels, CRFs effectively smooth segmentation boundaries and improve boundary precision. This process considers the similarity of neighboring pixels, preserving details while reducing noise interference. Despite the strong pixel-level performance demonstrated by these algorithms, their lack of explicit spatial constraints and instance localization mechanisms often leads to inaccuracies when dealing with small, overlapping targets. This limitation becomes particularly evident in scenarios involving densely packed sperm instances, where accurate distinction between overlapping instances is critical.

To overcome the limitations of existing top-down and bottom-up approaches, this paper proposes a sperm morphology parsing model that integrates instance segmentation and semantic segmentation, aiming to enhance instance distinction and part-level parsing accuracy. The instance segmentation branch generates explicit instance boundaries, enabling accurate differentiation of overlapping and densely packed sperm instances, thereby establishing a global structural constraint and effectively mitigating the incomplete parsing issues caused by the lack of global consistency in bottom-up approaches. Meanwhile, the semantic segmentation branch directly parses sperm subparts from feature maps without relying on detection boxes, providing finer-grained segmentation results. By fusing the outputs of both branches, the model achieves comprehensive parsing by capturing global instance-level information while preserving fine-grained part-level details.

### 2.2. Measurement Accuracy Enhancement

Existing studies primarily focus on methods for measuring sperm morphology [27,28,33,34,35,36]. However, these measurements rely on segmented low-resolution images, where the boundaries of non-stained, motile sperm often appear blurred under low magnification. Consequently, measuring sperm morphology parameters in low-resolution images presents significant challenges, making effective measurement accuracy enhancement essential for accurate morphological analysis. Current methods for improving measurement accuracy primarily focus on image enhancement techniques, which can be categorized into traditional image enhancement, super-resolution reconstruction, and multi-frame image fusion. Traditional image enhancement techniques, such as Contrast Limited Adaptive Histogram Equalization (CLAHE) [37,38], apply denoising and preprocessing to facilitate subsequent image segmentation. For example, CLAHE performs histogram equalization in localized regions of the image, introducing contrast-limiting mechanisms to avoid over-enhancement. It smooths transitions through bilinear interpolation, achieving a balance between local contrast enhancement and global consistency. Additional techniques, such as Laplacian sharpening and wavelet-based denoising, are also commonly employed to enhance image features. Super-resolution reconstruction techniques, such as Super-Resolution Convolutional Neural Network (SRCNN) [29] and Enhanced Super-Resolution Generative Adversarial Network (ESRGAN) [30], use deep learning or traditional image reconstruction algorithms to convert low-resolution images into high-resolution ones, restoring fine details. SRCNN [29] employs a three-layer convolutional network for super-resolution reconstruction. It first upsamples the low-resolution image to the target size using bicubic interpolation, extracts shallow features through the first convolutional layer, maps these features non-linearly through the second convolutional layer, and finally reconstructs the high-resolution image through the third convolutional layer. The model is trained by minimizing the mean squared error (MSE) to improve reconstruction quality iteratively. ESRGAN [30], a generative adversarial network for super-resolution, enhances image quality by refining the generator, discriminator, and loss functions. Its generator adopts a Residual-in-Residual Dense Block (RRDB) structure, incorporating dense connections and multi-level residual blocks to improve feature extraction. The discriminator uses a Relativistic Average Discriminator (RaGAN) to better capture differences between generated and real images, while an improved perceptual loss function is introduced to enhance visual quality by focusing on high-level feature differences. ESRGAN generates high-resolution images with richer details and greater realism. However, the challenges of applying super-resolution techniques to sperm morphology assessment lie in their long processing times and high dependency on training data, limiting their suitability for real-time sperm analysis. Multi-frame image fusion techniques, such as Sparse Coding Super-Resolution (SCSR) and Projection Onto Convex Sets (POCS), synthesize high-resolution images by registering and fusing multiple low-resolution images of the same scene. SCSR [39] utilizes sparse representation theory to match sparse features across multiple frames, achieving image fusion and reconstruction. POCS [40] iteratively projects pixel values onto convex sets defined by constraints, gradually generating high-resolution images. Despite visual improvements achieved by traditional image enhancement techniques, they cannot fundamentally resolve the information loss and feature blurring inherent in low-resolution images. Super-resolution reconstruction and multi-frame image fusion techniques require substantial computational resources and long processing times, making them unsuitable for real-time analysis in dynamic scenarios such as motile sperm morphology assessment.

To address the inherent challenges posed by low-resolution images, including information loss and boundary ambiguity, while ensuring real-time applicability, this paper proposes a measurement accuracy enhancement method based on statistical analysis and signal processing. By leveraging the periodic characteristics of sperm’s helical motion, the method accurately identifies true peaks within each cycle, effectively mitigating the impact of noise and local fluctuations, thereby preserving essential features. The interquartile range (IQR) method is employed to eliminate outliers, reducing random errors and ensuring robust and consistent measurement results. Gaussian filtering further smooths the boundaries, alleviating issues caused by noise and boundary irregularities, enhancing continuity and clarity. Lastly, robust boundary correction iteratively refines the detected boundaries to better align with the true morphology of sperm, improving measurement accuracy. With its lightweight design and low computational complexity, the proposed method avoids reliance on high-resolution reconstruction or multi-frame fusion, enabling efficient real-time analysis in dynamic scenarios, thus overcoming the limitations of traditional methods that require significant computational resources and longer processing times.

## 3. Methods

To achieve quantitative morphological measurements of sperm parts, including the head, midpiece, and tail, this study developed a novel non-stained sperm morphology measurement method. The proposed method comprises two key components: (1) a multi-scale part parsing network integrating semantic and instance segmentation to enable precise instance-level parsing of sperm morphology, and (2) a measurement accuracy enhancement method based on statistical analysis and signal processing to mitigate measurement errors caused by the reduced resolution of non-stained sperm images. By systematically combining these two components, the proposed method ensures accurate, automated, and non-invasive sperm morphology measurement. The following sections provide detailed descriptions of these two components.

### 3.1. Multi-Scale Part Parsing Module Integrating Semantic and Instance Segmentation

**Figure 2:**
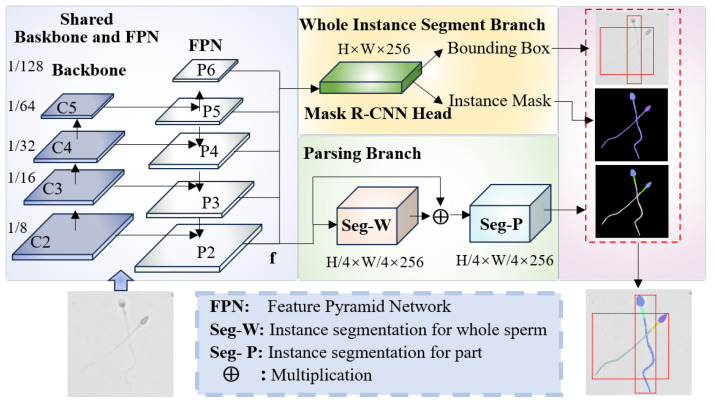
Architecture of the proposed multi-scale part parsing model integrating semantic and instance segmentation. The input image is first processed through the convolutional backbone and FPN within the multi-scale part parsing module, generating fused features extracted from different hierarchical levels. These fused features are utilized by two branches: one branch applies an instance segmentation model to produce instance masks for each sperm, while the other branch performs semantic segmentation to generate fine-grained features for different sperm parts. Finally, an alignment operation merges the outputs from both branches, resulting in instance-level parsing features for individual sperm.

Figure 2 illustrates the overall architecture of the proposed multi-scale part parsing model integrating semantic and instance segmentation. The input image is first processed through the convolutional backbone and Feature Pyramid Network (FPN) [41] within the multi-scale part parsing module, generating fused features extracted from different hierarchical levels. The fused features are then utilized by two parallel branches: one branch employs an instance segmentation model to generate instance masks for each sperm, while the other branch performs semantic segmentation to produce fine-grained segmentation features for different sperm parts. Finally, an alignment operation combines the results from both branches, yielding instance-level parsing features for individual sperm.

The multi-scale part parsing model consists of two branches: a standard Mask R-CNN branch, responsible for generating sperm instance segmentation masks ($S_{inst}$) and bounding boxes ($B_{box}$), and a sperm parsing and classification branch. The sperm parsing branch is a fine-grained semantic segmentation network designed to further segment different parts of each sperm instance. By fusing the outputs of these two branches, precise parsing of sperm and its components is achieved. Specifically, the module utilizes two branches to collaboratively perform sperm segmentation and part parsing. Features at different levels $P_{2}$, $P_{3}$, $P_{4}$, $P_{5}$ are first extracted from a Feature Pyramid Network (FPN). To account for the scale differences among feature maps, $P_{3}$, $P_{4}$, and $P_{5}$ are upsampled to match the resolution of $P_{2}$. The upsampled features are then concatenated with $P_{2}$ and fused through a 1 × 1 convolutional layer to generate a fused feature map *f*, which serves as the input for subsequent segmentation tasks.

(1) $${\tilde{P}}_{i}=\mathrm{Up}\left(P_{i}\right),\;i\in \left\{3,4,5\right\}$$

(2) $$f={\mathrm{Conv}}_{1\times 1}\left(\mathrm{Concat}\left(P_{2},{\tilde{P}}_{3},{\tilde{P}}_{4},{\tilde{P}}_{5}\right)\right),\;i\in \left\{3,4,5\right\}$$

The upsampling process is described in Equation (1), where *Up* represents the upsampling function. Equation (2) defines the process of concatenating and fusing these features through a 1 × 1 convolutional layer.

Secondly, the instance segmentation branch, based on the standard Mask R-CNN, processes the fused feature *f* to generate sperm instance segmentation masks $S_{inst}$ and bounding boxes $B_{box}$. Simultaneously, the fine-grained semantic segmentation branch performs an initial whole sperm segmentation followed by part-level parsing. A two-step method is employed for sperm part parsing, as outlined below:

Step 1: Whole Segmentation Phase

**Figure 3:**
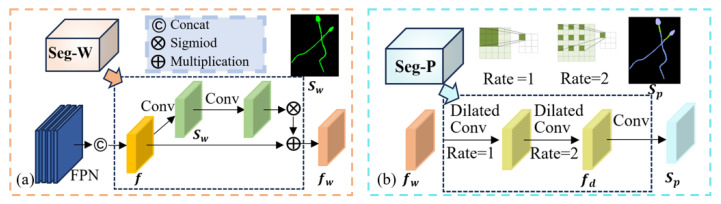
Whole segmentation phase and part segmentation phase. (**a**) Whole segmentation phase. (**b**) Part segmentation phase.

In this phase (see Figure 3a), the fused feature *f* undergoes feature transformation through a 1 × 1 convolution to generate a coarse segmentation mask $S_{w}$, which represents the resolution size of the features. Subsequently, $S_{w}$ is used as input for another 1 × 1 convolution to further refine the features in preparation for generating accurate masks. The output of the second convolution is then activated using the Sigmoid function. The result of the Sigmoid activation is element-wise multiplied with the original fused feature *f*. This mask modulation enhances the model’s focus on sperm-specific features by emphasizing sperm regions in the image while suppressing non-sperm areas. The output is a sperm instance segmentation feature $f_{w}$, which is used for further sperm segmentation tasks.

(3) $$S_{w}={\mathrm{Conv}}_{1\times 1}\left(f\right)$$

(4) $$f_{w}=\sigma \left({\mathrm{Conv}}_{1\times 1}\left(S_{w}\right)\right)⊙f$$

Here, σ represents the Sigmoid activation function, and ⊙ denotes the element-wise multiplication operation.

Step 2: Part Segmentation Phase

In this phase (see Figure 3b), the whole segmentation feature $f_{w}$ is further processed using a cascade of dilated convolutional layers. By employing different dilation rates, the receptive field is effectively expanded without introducing additional computational overhead or increasing the number of parameters. This allows the network to capture a broader range of contextual information, resulting in more fine-grained segmentation features $f_{p}$ for each sperm part. Subsequently, a 1 × 1 convolution is applied to $f_{p}$ to generate the part segmentation mask $S_{p}$, where $S_{p}$ represents the resolution size of the features.

(5) $$f_{p}=\mathrm{Dilated}\;{\mathrm{Conv}}_{3\times 3}\;f_{w},\;DR=\left\{1,2\right\}$$

(6) $$S_{p}={\mathrm{Conv}}_{1\times 1}\left(f_{p}\right)$$

Finally, the outputs of the two branches are fused using an alignment operation, combining the part mask $S_{p}$ generated by semantic segmentation with the instance mask $S_{inst}$ from instance segmentation. This ensures accurate segmentation of both the sperm and its parts, as expressed by the following equation:

(7) $$S_{\mathrm{final}}=S_{\mathrm{inst}}⊙S_{p}$$

where $S_{final}$ represents the final segmentation result of the sperm instance and its parts.

This method leverages the precision of instance segmentation and the global contextual information provided by semantic segmentation, achieving high-accuracy parsing of sperm and its parts. Throughout the segmentation process, the model’s total loss function, $L_{total}$, is composed of the instance segmentation loss, $L_{inst}$, and the part segmentation loss, $L_{part}$, as follows:

(8) $$L_{\mathrm{total}}=\lambda_{\mathrm{inst}}L_{\mathrm{inst}}+\lambda_{\mathrm{part}}L_{\mathrm{part}}$$

where $\lambda_{inst}$ and $\lambda_{part}$ are weighting coefficients used to balance the contributions of instance segmentation and part segmentation.

The instance segmentation loss, $L_{inst}$, follows the standard Mask R-CNN loss, comprising the bounding box regression loss $L_{bbox}$, mask loss $L_{mask}$, and classification loss $L_{cls}$:

(9) $$L_{\mathrm{inst}}=L_{\mathrm{bbox}}+L_{\mathrm{mask}}+L_{\mathrm{cls}}$$

The part segmentation loss, $L_{part}$, is primarily calculated using binary cross-entropy loss, which measures the error between the generated part mask $S_{p}$ and the ground truth part mask $S_{gt}$:

(10) $$L_{\mathrm{part}}=-\frac{1}{N}\sum_{i=1}^{N}\left[{\tilde{S}}_{i}\log S_{i}+\left(1-{\tilde{S}}_{i}\right)\log\left(1-S_{i}\right)\right]$$

The combination of the whole segmentation phase and the part segmentation phase constitutes a coarse-to-fine segmentation strategy, ensuring both efficient global localization and precise local segmentation.

### 3.2. Measurement Accuracy Enhancement Method Based on Statistical Analysis and Signal Processing

To address the measurement errors caused by the reduced resolution of non-stained sperm images, a measurement accuracy enhancement method based on statistical analysis and signal processing is proposed. This method aims to mitigate the errors in sperm morphology measurements under low-resolution conditions and correct the deviations introduced by noise and blurred boundaries.

This study enhances the measurement accuracy of all required parameters following sperm morphology segmentation. These parameters include sperm head parameters (e.g., the length, width, and ellipticity of the fitted rectangle), midpiece parameters (e.g., length, width, and rotational angle), and tail parameters (e.g., curvature and folding). According to the WHO manual, the degree of progressive motility of sperm is correlated with pregnancy rates, with sperm exhibiting a velocity > 25μm/s at 37 °C classified as rapidly progressive. Under a frame rate of 60 frames per second (fps), sperm with such velocity can complete multiple spiral motions within 2 s. Therefore, in this study, all required measurements for sperm morphology parameters are collected from the 120 frames captured over this 2-s period, forming the dataset *X* for each morphological parameter.

(11) $$X=\left\{x_{1},x_{2},\ldots ,x_{i}\right\},\;i\in \left(1,120\right)$$

where $x_{i}$ represents the measurement data of a specific parameter (e.g., head length, head width, midpiece length, etc.) at the *i*-th frame.

**Figure 4:**
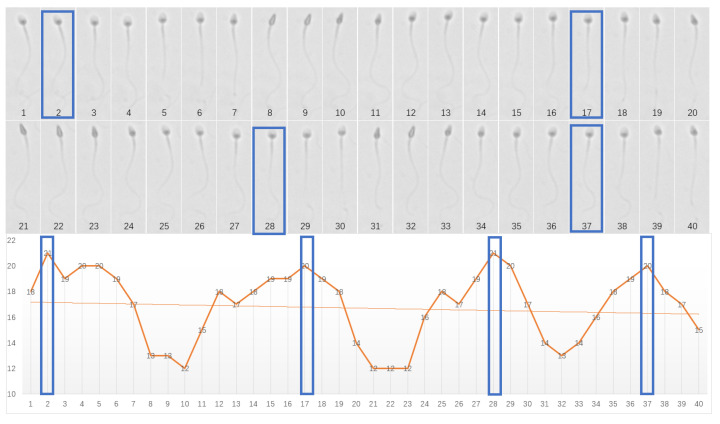
Analysis of sperm head width during spiral motion across consecutive frames. The horizontal axis represents the frames per second (fps), and the vertical axis denotes the sperm head width in pixels. Overall, the sperm head width exhibits a consistent pattern of variation corresponding to the spiral motion, reflecting the periodic nature of its changes.

During the spiral motion of sperm, highly active sperm rotate or oscillate at a frequency of approximately 3.7 Hz (about 3.7 rotations per second) under specific conditions. Consequently, sperm morphological parameters (e.g., width, length, etc.) exhibit periodic fluctuations, where each period generates a local maximum. Due to this periodic spiral motion, head parameters typically display a trend of gradually increasing from a low value to a peak and then decreasing, forming a cyclic pattern of fluctuations (see Figure 4). Unlike simple sorting methods that directly identify the maximum value, the local maximum extraction method can more accurately identify the peak of each cycle, avoiding misjudgments caused by noise or minor local variations. However, relying solely on individual local maxima may introduce false signals, especially when periodic noise is present in the signal. To address this, after identifying local maxima, neighboring points are further extracted to form a local maximum region. To retain contextual information around each local maximum, a window size *k* is defined, extending *k* points to the left and right of the local maximum. This results in a region of total size 2*k* + 1, which includes the local maximum point and its *k* adjacent points on both sides. Using this method, the local maxima and their surrounding neighborhood information for each cycle are preserved and integrated into a new dataset. The new dataset, denoted as $X^{\prime }$, is expressed as follows:

(12) $$X^{\prime }={\left\{X_{i}^{\prime }\right\}}_{i=1}^{j}$$

where ${X_{i}}^{\prime }$$=\{x_{i-k},x_{i-k+1}\ldots x_{i+k}\}$ represents the local maximum region for the *i*-th cycle, *j* is the total number of cycles, and $x_{i}$ is the local maximum within the cycle.

Subsequently, the dataset $X^{\prime }$ is processed using the interquartile range (IQR) method to filter out outliers, ensuring the stability and reliability of the measured data. The IQR is calculated as follows:

(13) $$X^{\prime \prime }\in \left[Q_{1}-1.5*\mathrm{IQR},Q_{3}+1.5*\mathrm{IQR}\right]$$

Here, $X^{\prime \prime }$ denotes the dataset after outlier removal.

To reduce local fluctuations and noise in the measured data, the filtered dataset $X^{\prime \prime }$ is further processed using Gaussian filtering, producing a smoothed dataset $X^{\prime \prime \prime }$. Gaussian filtering achieves smoothing by applying a weighted average to each data point and its neighboring points, with weights determined by a Gaussian distribution. Points farther from the center are assigned smaller weights. Sperm measurement data are prone to local noise interference, such as low image resolution or blurred boundaries, leading to unstable fluctuations in certain measurements. By applying weighted processing to each data point and its neighbors, Gaussian filtering reduces random errors and local fluctuations, ensuring that the filtered data better reflect the true morphology of the sperm.

The Gaussian filtering is defined as follows:

(14) $$X_{i}^{\prime \prime \prime }=\sum_{j=-s}^{k}G\left(j\right)*X_{i+j}^{\prime \prime }$$

where *s* is the size of the filtering window, determining the number of neighboring points included in the smoothing process. $G\left(j\right)=\frac{1}{\sqrt{2\pi \sigma^{2}}}\exp\left(-\frac{j^{2}}{2\sigma^{2}}\right)$ is the Gaussian weight for the *j*-th data point. σ is the standard deviation of the Gaussian function, controlling the degree of smoothing. Finally, the maximum value is extracted from the non-outlier dataset $X_{i}^{\prime \prime \prime }$ as follows:

(15) $$Y=\max\left(x_{i}|x_{i}\in X_{i}^{\prime \prime \prime }\right)$$

## 4. Experiments and Results

### 4.1. Datasets, Implementation Details and Evaluation Metric

In this study, we constructed a novel dataset focusing on sperm part segmentation, aiming to accurately identify individual sperm instances and finely segment their components (i.e., head, midpiece, and tail). All images were manually annotated by professionals, with each sperm part assigned a unique instance ID and label. The dataset comprises 300 images with a resolution of 1200 × 900, with an average of 15–30 sperm instances per image. To ensure effective model training and evaluation, 80% of the dataset was randomly allocated to the training set, while the remaining 20% was reserved for testing.

The proposed model was implemented using PyTorch (1.13.1) based on the mmdetection framework. The backbone network is ResNet50 pre-trained on ImageNet, combined with an FPN to extract multi-scale features, with the output channel size set to 256 for each level. The model consists of two branches: the instance segmentation branch adopts a standard Mask R-CNN to perform sperm instance segmentation and bounding box regression, while the semantic segmentation branch employs dilated convolutions and multi-scale feature fusion to achieve fine-grained parsing of the sperm head, midpiece, and tail. The model supports single-class sperm instance segmentation (sperm) and four-class part segmentation (head, midpiece, and tail). During training, the input image resolution is set to 1200 × 900, and the images are normalized using the ImageNet mean and standard deviation (mean = [0.485, 0.456, 0.406], std = [0.229, 0.224, 0.225]). Additionally, random horizontal flipping is applied as a data augmentation strategy to improve the model’s generalization capability. The batch size is set to 4, and the AdamW optimizer is used, with an initial learning rate of 0.0001. The model is trained for 50 epochs, with the learning rate decayed by a factor of 0.1 at epochs 30 and 40. For inference, multi-scale testing and flipping augmentation are employed to enhance the final segmentation accuracy. The total training time on an NVIDIA RTX 4090 (NVIDIA, Santa Clara, CA, USA) is approximately 10 h.

To comprehensively evaluate the performance of the proposed network, we adopted both global- and instance-level metrics to analyze the segmentation performance from different perspectives. First, mean intersection over union (mIoU) was used as the key metric for assessing global semantic segmentation performance. mIoU measures the segmentation accuracy across all classes by calculating the ratio of the overlap between predicted and ground-truth segmentation to their union, providing a quantitative measure of overall performance. This global evaluation helps assess the model’s consistency across different sperm instances. Second, we employed part-based average precision (AP) and percentage of correctly parsed semantic parts (PCP) to measure instance-level segmentation accuracy. Instance-level metrics provide a detailed assessment of the model’s segmentation performance on individual sperm instances, ensuring a focus on both overall performance and the accuracy of individual sperm segmentation. Specifically:

- ${AP}_{50}^{p}$ reflects the average precision at an IoU threshold of 0.5.
- ${PCP}_{50}$ measures the percentage of correctly parsed parts at the same IoU threshold.
- ${AP}_{vol}^{p}$ computes the average precision across IoU thresholds ranging from 0.1 to 0.9 in incremental steps, providing a comprehensive evaluation of the model’s performance under varying degrees of overlap.

### 4.2. Comparisons with State-of-the-Art Models

**Figure 5:**
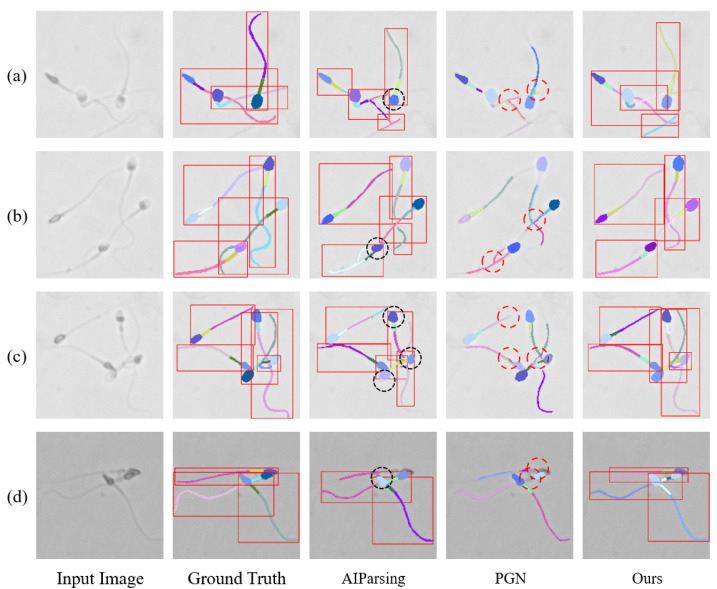
Qualitative comparison of instance-level sperm target parsing results. (**a**–**d**) represent the processing results of different example images using various methods. AIParsing (a top-down approach) shows a strong dependency on bounding boxes, resulting in the exclusion of contextual information outside the bounding box. PGN (a bottom-up approach) struggles with instance differentiation, failing to separate overlapping sperm parts. In contrast, the proposed network achieves the best predictions by combining global feature information for instance differentiation with local detail information that is independent of bounding box localization.

To evaluate the performance of sperm part segmentation, we compared the proposed network with several state-of-the-art instance-level part segmentation models, including top-down methods (AIParsing, Parsing R-CNN, CE2P, HRNet) and bottom-up methods (PGN, Graphonomy, DeepLab V3+). All methods were tested on our collected dataset, and the qualitative and quantitative comparison results are summarized in Figure 5 and Table 1.

Figure 5 illustrates the qualitative comparison of instance-level sperm segmentation results between our proposed model and state-of-the-art methods, including PGN, AIParsing, and our model. Among these, PGN demonstrates the poorest performance in instance-level sperm parsing. As a classical bottom-up parsing model, PGN effectively identifies and parses targets but fails to distinguish overlapping sperm instances (see Figure 5, red circles). Clearly, PGN struggles in cases where multiple sperm heads overlap or are closely packed together, leading to incorrect merging of instances (see Figure 5c, black circles). AIParsing, a top-down solution, performs better at separating different instances but heavily relies on bounding boxes. This dependency leads to the exclusion of contextual information outside the bounding box (see Figure 5, red circles).

In contrast, our proposed parsing model produces segmentation results that closely match the ground truth, even in challenging scenarios with non-uniform backgrounds and slight contrast variations (see Figure 5d). This better performance stems from two key aspects of our network. First, it leverages global feature information to achieve effective instance differentiation. Unlike PGN and AIParsing, our model can maintain high segmentation accuracy by capturing contextual information across the entire image, rather than relying solely on local or bounding-box-constrained regions. Second, its coarse-to-fine segmentation module allows for a more focused segmentation of specific subregions by initially coarsely localizing each sperm cell, followed by finer segmentation of individual parts. These features enable our model to outperform existing solutions in accurately parsing sperm instances and their components, especially in cases with overlapping instances or varying backgrounds.

#### 4.2.1. Performance on Global Parsing Metrics

As shown in Table 1, the proposed method achieved the highest mIoU score of 63.2%, significantly outperforming other methods. When using the same backbone, ResNet-50, the proposed method achieved the best mIoU score, surpassing the second-best AIParsing by 5.1%. AIParsing employs an anchor-free detector (FCOS) [42] instead of anchor-based detectors like Mask R-CNN, enhancing detection performance with strong instance differentiation capabilities. However, AIParsing relies on global features for coarse predictions followed by gradual refinement, often neglecting small-scale information. This issue is particularly pronounced when using large convolutional kernels or fewer sampling points, where features of small structures like sperm heads may be smoothed or weakened, leading to suboptimal segmentation performance (as indicated by the red areas in Figure 5b for AIParsing).

#### 4.2.2. Performance on Instance-Level Parsing Metrics

For instance-level parsing metrics, AIParsing demonstrates notable advantages among top-down methods, achieving ${AP}_{vol}^{p}$(45.2) and ${AP}_{50}^{p}$(55.8). Using ResNet-50 as the backbone, AIParsing outperformed CE2P (which uses ResNet-101) by 3.9% on ${AP}_{vol}^{p}$ and 6.0% on ${AP}_{50}^{p}$. The advantage lies in AIParsing’s parallel detection and parsing modules, which avoid parsing errors caused by detection issues—a limitation in CE2P, where parsing heavily depends on serially linked detection results. However, AIParsing’s reliance on global features for coarse predictions leads to the neglect of fine-scale information, particularly for small subcellular structures like sperm heads, as highlighted earlier. In contrast, the proposed model outperforms AIParsing significantly, achieving a margin of 14.1% ${AP}_{vol}^{p}$/10.4% ${AP}_{50}^{p}$/6.7% ${PCP}_{50}$ when both use ResNet-50 as the backbone. This superior performance is largely attributed to the dual-branch segmentation design, integrating semantic and instance segmentation. The semantic segmentation branch’s fine-grained segmentation network avoids reliance on bounding boxes, enabling more precise segmentation of small subcellular structures, further enhancing overall segmentation accuracy.

#### 4.2.3. Processing Speed

For processing speed, the proposed method achieved an average speed of 17.5 FPS on 300 validation images, measured on a single NVIDIA RTX A6000 GPU. This ensures high computational efficiency while maintaining high segmentation accuracy. By comparison, Parsing R-CNN achieved a faster speed of 19.9 FPS but with significantly lower segmentation accuracy (54.2% ${AP}_{50}^{p}$). On the other hand, AIParsing and Graphonomy demonstrated slower processing speeds of 11.8 FPS and 11.5 FPS, respectively, indicating limited efficiency in practical applications. Given the complexity of non-stained sperm segmentation tasks involving intricate morphological structures and part parsing, high segmentation accuracy is crucial for reliable sperm morphology analysis. While prioritizing speed might yield short-term performance gains, sacrificing segmentation accuracy can result in segmentation failures and misjudgment of sperm morphology. The proposed method achieves a balanced trade-off between accuracy and processing speed, with 66.2% ${AP}_{50}^{p}$, ensuring detailed sperm morphology parsing while maintaining real-time processing capabilities. This balance makes the method well suited for applications requiring both accuracy and efficiency.

### 4.3. Effectiveness of Backbone Network Selection

To validate the rationality of using ResNet50 as the backbone network, we conducted ablation experiments by comparing ResNet34, ResNet50, and ResNet101. The goal was to evaluate the impact of different backbones on segmentation accuracy and inference efficiency, as these networks have varying feature extraction capabilities and computational complexities. The experiments confirmed that ResNet50 strikes a balance between accuracy and efficiency, making it a reasonable choice for this task.

To ensure a fair comparison, we maintained consistent model components across experiments and measured multiple metrics, including mIoU, ${AP}_{vol}^{p}$, ${AP}_{50}^{p}$, ${PCP}_{50}$, FPS, parameter count, and floating point operations per second (FLOPs). Table 2 summarizes the comparison results across different backbones. As shown in the table, although ResNet34 achieved the fastest inference speed (20.3 FPS), its feature extraction capability was relatively weak, resulting in lower accuracy (mIoU = 60.1%). Consequently, it failed to meet the accuracy requirements of sperm morphology analysis. In contrast, ResNet101 improved accuracy slightly (mIoU = 64.1%) but suffered from significantly lower inference speed (11.2 FPS) and higher computational cost, limiting its practical applicability in real-time scenarios. Compared to these two networks, ResNet50 achieved a good balance, obtaining 63.2% mIoU, 59.3% ${AP}_{vol}^{p}$, 66.2% ${AP}_{50}^{p}$, and an inference speed of 17.5 FPS, demonstrating both competitive accuracy and high practical usability.

As shown in Table 2, ResNet50 achieves the best balance between accuracy and inference efficiency. Although ResNet101 achieves slightly higher accuracy than ResNet50, the increased computational cost and slower inference speed make it unsuitable for real-time sperm morphology analysis. On the other hand, while ResNet34 offers faster inference, it lacks sufficient accuracy for precise morphology measurement. Therefore, ResNet50 is verified as the optimal backbone choice, offering both high segmentation accuracy and real-time performance, meeting the comprehensive requirements for sperm morphology analysis.

### 4.4. Quantification of Morphology Parameters

Based on the segmentation masks generated by the proposed network, a quantitative analysis of sperm morphology was conducted. Parameters of the head and midpiece were calculated using elliptical and rectangular fitting methods, providing measurements such as length, width, and ellipticity. For the tail, the Steger automatic morphology measurement method was applied to accurately extract parameters such as length, width, and angle. Table 3 summarizes the quantitative metrics for each sperm part.

To evaluate the performance of the proposed morphology measurement method based on statistical analysis, an additional experiment was designed, where the morphological characteristics of 50 sperm samples were measured and compared against manual baseline methods. In this experiment, the head’s length, width, and ellipticity, the midpiece’s length, width, and angle, and the tail’s length, width, and angle were used as quantitative comparison metrics. Baseline data were obtained by manually magnifying and precisely annotating the original images to minimize human error.

The relative error (Errors) was used to represent the difference between the measured values and the baseline values, calculated as follows:

(16) $$Errors=\frac{V_{M}-V_{GT}}{V_{GT}}\times 100\%$$

where $V_{M}$ represents the measured value, and $V_{GT}$ represents the manually annotated ground truth baseline value.

**Figure 6:**
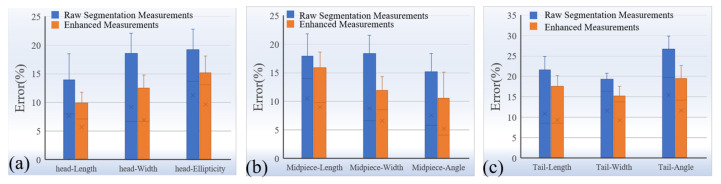
Quantitative comparison of measurement errors for sperm head, midpiece, and tail parameters. (**a**) Quantitative comparison of relative errors for sperm head parameters, including length, width, and ellipticity. (**b**) Quantitative comparison of relative errors for sperm midpiece parameters, including length, width, and angle. (**c**) Quantitative comparison of relative errors for sperm tail parameters, including length, width, and angle.

The comparison results are summarized in Figure 6a–c. Before applying the proposed measurement accuracy enhancement method, the measurement errors of sperm morphological parameters were substantial and exhibited significant instability. Specifically, the relative errors for head parameters—length, width, and ellipticity—were 13.91%, 18.57%, and 19.21%, respectively. For midpiece parameters, the relative errors for length, width, and angle were 17.89%, 18.36%, and 15.14%, respectively. Tail parameters showed the highest relative errors, with length, width, and angle errors of 21.61%, 19.3%, and 26.72%, respectively. These high errors primarily stemmed from blurred boundaries in low-resolution images, noise interference, and periodic fluctuations caused by sperm spiral motion, leading to poor accuracy and consistency in the measurements.

In contrast, the proposed measurement accuracy enhancement method, based on statistical analysis and signal processing, effectively mitigated these issues through multi-stage processing involving local maximum extraction, outlier removal, and Gaussian filtering. The experimental results showed that after processing, the relative errors for head parameters—length, width, and ellipticity—were reduced to 9.87%, 12.46%, and 15.16%, representing reductions of 29.0%, 32.9%, and 21.1%, respectively. For midpiece parameters, the relative errors for length, width, and angle decreased to 15.87%, 11.93%, and 10.52%, corresponding to reductions of 11.3%, 35.0%, and 30.5%, respectively. Tail parameters also saw significant improvements, with length, width, and angle errors reduced to 17.59%, 15.25%, and 19.47%, achieving reductions of 18.6%, 21.0%, and 27.2%, respectively.

These substantial improvements were achieved through multiple optimization measures in the proposed method. First, the local maximum extraction method leveraged the periodic nature of sperm spiral motion to accurately identify the true peaks in each cycle, avoiding false signals caused by noise or local fluctuations. Second, outlier removal using the interquartile range (IQR) method effectively eliminated outliers caused by boundary blurring or noise interference, ensuring data stability and reliability. Finally, Gaussian filtering smoothed data fluctuations, reducing random errors and enabling the filtered measurements to better reflect the true morphology of the sperm. The method demonstrated particularly high accuracy and consistency in complex morphological parameters significantly affected by motion, such as tail angle and midpiece width. These results highlight that the proposed method, by integrating periodicity-based analysis, outlier removal, and data smoothing techniques, significantly reduced the relative errors of sperm morphological parameter measurements. It also improved measurement consistency and reliability, providing an effective solution for automated sperm morphology measurement under low-resolution imaging conditions.

## 5. Conclusions and Future Work

This study presents a novel automated and non-invasive method for sperm morphology measurement, addressing key challenges in sperm segmentation and parameter measurement under low-resolution, non-stained imaging conditions. By integrating a multi-scale part parsing network with measurement accuracy enhancement techniques, the proposed method achieves precise segmentation of individual sperm instances and their components, including the head, midpiece, and tail. The incorporation of statistical analysis and signal processing further improves measurement accuracy, effectively mitigating errors caused by noise, blurred boundaries, and the periodic motion of sperm. Experimental results demonstrate that the proposed method enables automated, non-invasive measurement of morphological parameters for multiple sperm targets, offering a reliable and efficient solution for sperm morphology analysis.

Despite the encouraging results obtained, there are still several challenges that require further research. First, we aim to explore virtual staining techniques to enhance the contrast of unstained sperm images. Traditional staining methods improve the visibility of sperm structures but are invasive and unsuitable for real-time or repeated measurements. To overcome this limitation, we plan to develop a deep-learning-based virtual staining model using generative adversarial networks (GANs) or conditional GANs (cGANs). This model will generate synthetic stained images from unstained inputs, improving segmentation accuracy while maintaining the non-invasive nature of the approach. By training on datasets collected under diverse imaging conditions, we expect to enhance the model’s generalizability, ensuring applicability across different microscopy setups. Second, we plan to develop a knowledge-graph-based framework for abnormal sperm detection. Current methods often rely on handcrafted rules or simple image features, leading to inconsistent results in complex cases. By constructing a knowledge graph that explicitly represents relationships between sperm morphological features and abnormalities, we can enable more robust and interpretable inference. A graph neural network (GNN) will be employed to reason over the constructed graph and infer abnormality types based on extracted features from segmented sperm instances. This approach is expected to improve accuracy, reduce reliance on large annotated datasets, and enhance the interpretability of abnormal sperm detection. In summary, while the proposed stained-free sperm morphology measurement method offers promising capabilities for real-world applications, addressing these challenges will further enhance its robustness, versatility, and clinical utility. Future work on virtual staining and knowledge-graph-based abnormal sperm detection will contribute to making non-invasive sperm analysis more accurate and reliable, facilitating broader adoption in clinical and research contexts. 

## Figures and Tables

**Table 1:** Performance comparison of segmentation methods.

Method	Backbone	mIOU	${\mathrm{AP}}_{\mathrm{vol}}^{p}$	${\mathrm{AP}}_{50}^{p}$	${\mathrm{PCP}}_{50}$	FPS
**Top-down:**						
AIParsing [22]	ResNet-50	58.1	45.2	55.8	54.5	11.8
Parsing R-CNN [31]	ResNet-50	53.5	43.7	54.2	52.4	**19.9**
CE2P [23]	ResNet-101	57.5	41.3	49.8	-	0.31
HRNet [24]	HRNetV2-W48	55.5	40.9	47.6	-	0.43
**Bottom-up:**						
PGN [20]	ResNet-101	55.7	24.6	32.1	29.8	7.8
Graphonomy [32]	ResNet-101	56.3	-	-	-	11.5
DeepLab V3+ [26]	Xception	55.9	-	-	-	15.2
Ours	ResNet-50	**63.2**	**59.3**	**66.2**	**61.2**	17.5

**Table 2:** Comparison results of different backbone networks.

Backbone	mIoU (%)	${\mathrm{APp}}_{\mathrm{vol}}$ (%)	${\mathrm{APp}}_{50}$ (%)	${\mathrm{PCP}}_{50}$ (%)	FPS	Parameters (M)	FLOPs (G)
ResNet34	60.1	56.8	62.1	57.5	20.3	21.3	3.6
ResNet50	63.2	59.3	66.2	61.2	17.5	25.6	4.1
ResNet101	64.1	60.5	67.0	62.7	11.2	44.7	7.9

**Table 3:** Quantitative features for each part of observed sperm.

Sample	ID	Head			Midpiece			Tail		
		**Length (μm)**	**Width (μm)**	**Ellipticity (AU)**	**Length (μm)**	**Width (μm)**	**Angle (AU)**	**Length (μm)**	**Width (μm)**	**Angle (^∘^)**
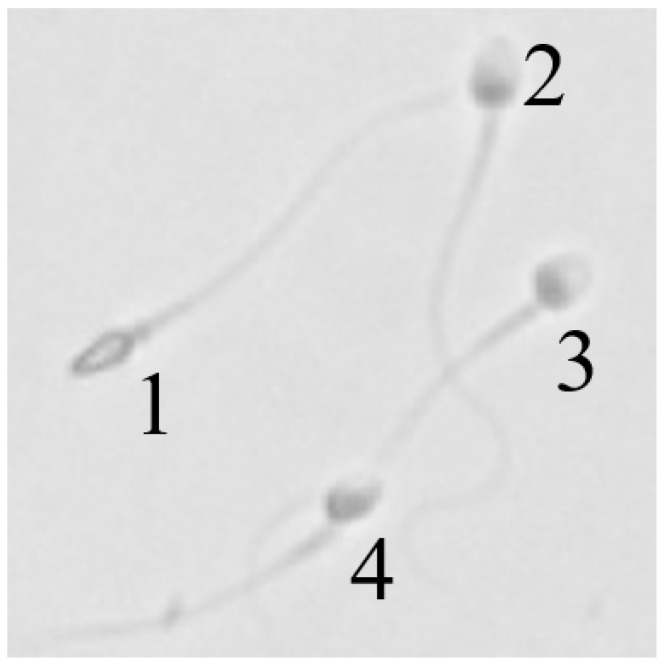	1	4.28	2.15	1.86	3.95	0.64	0.21	21.65	0.58	18.86
	2	4.51	2.85	1.85	4.01	0.58	8.48	27.56	0.49	23.15
	3	4.48	2.65	1.78	4.11	0.49	10.12	26.62	0.65	18.62
	4	5.02	2.95	1.92	4.23	0.61	15.67	31.65	0.53	24.84

## Data Availability

The data presented in this study are available upon request from the corresponding author.
